# A dual catastrophe in the left main coronary artery: iatrogenic dissection and dislodgement of the fully deployed stent

**DOI:** 10.1186/s12872-026-05563-9

**Published:** 2026-02-07

**Authors:** Sefa Sural, Vedat Aslan, Gökhan Avcı

**Affiliations:** 1 Vocational School of Health Service, Toros University, Bahçelievler 1835 Sokak. No: 4 Yenişehir, Mersin, 33140 Turkey; 2 Department of Cardiology, Medical Park Hospital, Gazi Mustafa Kemal Bulvari. No 676. Mezitli, Mersin, Turkey

**Keywords:** Iatrogenic left main artery dissection, Coronary artery dissection, Stent dislodgement, Percutaneous coronary intervention

## Abstract

**Background:**

Iatrogenic left main coronary artery (LMCA) dissection and stent dislodgement are rare but potentially life-threatening complications of coronary angiography and percutaneous coronary intervention. Their simultaneous occurrence is extremely uncommon and poses significant technical challenges.

**Case presentation:**

A 62-year-old man with a history of hypertension developed LMCA dissection accompanied by chest pain during coronary angiography performed via the right radial approach, caused by non-coaxial catheter engagement. To stabilize the dissection, an additional femoral access was obtained. A drug-eluting stent implanted from the circumflex artery to the LMCA adhered to the balloon and migrated retrogradely, becoming lodged at the tip of the guiding catheter. Because retrieval attempts were unsuccessful, the dislodged stent was intentionally implanted into the right brachial artery to prevent distal embolization. The patient remained hemodynamically stable throughout the procedure and was discharged without complications.

**Conclusions:**

This case represents the first reported instance of simultaneous LMCA dissection and complete retrograde stent dislodgement successfully managed percutaneously without the need for surgical intervention. Prompt recognition, access modification, and careful device manipulation were essential for achieving a favorable outcome.

**Supplementary Information:**

The online version contains supplementary material available at 10.1186/s12872-026-05563-9.

## Background

Iatrogenic left main coronary artery (LMCA) dissection is a rare but potentially life-threatening complication that can occur during coronary angiography and interventional procedures if not promptly recognized and managed [1–3]. This condition is approximately twice as frequent during percutaneous coronary interventions (PCI) compared to diagnostic catheterization. The management strategy depends on the patient’s clinical stability and the extent of the dissection, ranging from conservative monitoring to PCI or coronary artery bypass grafting (CABG) [4].

In addition, stent dislodgement is another rare yet serious complication in interventional cardiology, which can lead to distal embolization or acute vessel occlusion. While such cases typically involve under-expanded stents, retrograde migration of a fully deployed stent is exceedingly uncommon [5].

In this case report, we present a unique scenario in which two distinct and rare complications—iatrogenic LMCA dissection and retrograde dislodgement of a fully deployed stent—occurred simultaneously. Both were successfully managed using percutaneous techniques without the need for surgical intervention. To the best of our knowledge, this is the first reported case in the literature where both complications were resolved percutaneously without the need for surgery.

## Case presentation

**Fig. 1:**
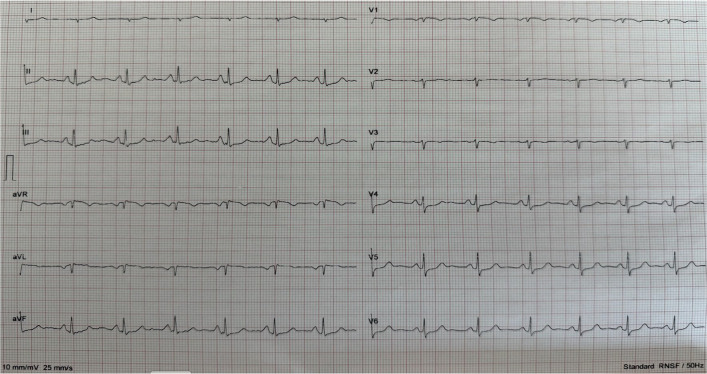
The initial electrocardiogram was taken upon admission

A 62-year-old male patient presented to the emergency department with syncope preceded by chest pain. Initial evaluation revealed an elevated troponin level of 2.03 µg/L (reference range: 0–0.16), supporting the diagnosis of non-ST elevation myocardial infarction (NSTEMI). The patient was subsequently admitted to the coronary intensive care unit. Baseline laboratory tests showed a mildly elevated hemoglobin level of 18.6 g/dL (reference range: 14–18), serum creatinine of 0.94 mg/dL, and an estimated glomerular filtration rate (eGFR) of 86.7 mL/min/1.73 m^2^. The initial electrocardiogram (Fig. 1) was reviewed and showed minor ST changes. Transthoracic echocardiography indicated preserved left ventricular systolic function with an estimated ejection fraction (EF) of 60%, and no significant valvular pathology was observed. On admission, heart rate was 75 bpm, and blood pressure was 90/50 mmHg, prompting initiation of intravenous fluid replacement due to hypotension.

The patient had a 20-year history of hypertension without diabetes mellitus. His regular medications included nebivolol 5 mg, olmesartan 20 mg, and doxazosin 4 mg. He stated that he forgot he had already taken his usual daytime dose of doxazosin and mistakenly retook it in the evening, resulting in duplicate dosing. He had no history of chronic obstructive pulmonary disease and was a former smoker.

**Fig. 2:**
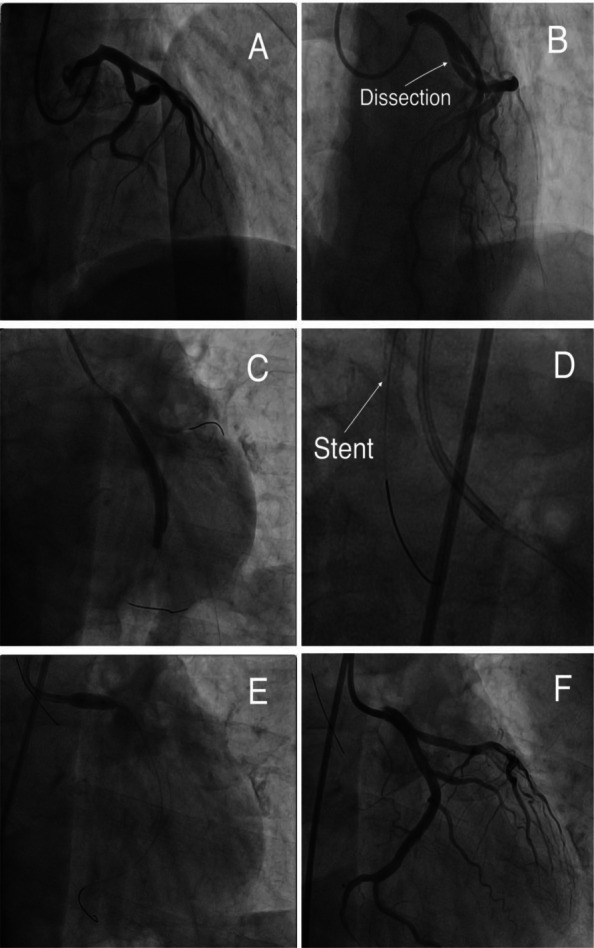
Illustration of coronary dissection and its management. **A** Non-coaxial engagement of the guiding catheter with the LMCA, preceding the dissection. **B** Angiographic visualization of the dissection flap extending into the CX. **C** Successful wiring and drug-eluting stent implantation into the dissected segment of the CX. **D** Proximal stent dislodgement: the fully deployed coronary stent recoiled and became lodged circumferentially around the tip of the guiding catheter, resembling a ring. **E** Optimization of the second stent deployed from LMCA to CX by high-pressure post-dilation using a 5.0 × 10 mm NC balloon at the ostium of the left main. **F** Final angiographic outcome demonstrating successful containment of the dissection and restoration of normal coronary flow. (LMCA = left main coronary artery; CX = circumflex coronary artery; NC = non-compliant)

Coronary angiography (CAG) was scheduled due to elevated troponin levels. Vascular access was established through the right radial artery.The right coronary artery (RCA) was visualized first and found to be normal. Subsequently, the left coronary system was targeted. Selective catheterization with a JL4 diagnostic catheter was unsuccessful. Given the elevated troponin and the anticipated need for intervention, imaging was continued using an EBU 6F 3.5 guiding catheter. However, coaxial engagement of the left main coronary artery (LMCA) could not be achieved with this catheter (Fig. 2A). Following contrast injection, an iatrogenic dissection was noted, extending from the LMCA ostium to the LAD–CX bifurcation, resulting in impaired distal coronary flow and chest pain (Fig. 2B). This was recognized as the first major complication, prompting immediate intervention.

A guidewire was urgently advanced from the LMCA into the circumflex artery (CX). As the dissection extended distally along the CX, stenting of this segment was performed. First, a 3.5 × 44 mm Supraflex (Sahajanand Medical Technologies, Surat, India) DES was successfully implanted (Fig. 2C). Then, a second 4.0 × 15 mm Xience PRO DES (Abbott Vascular, Santa Clara, U.S.A.) was deployed from the proximal and ostial segments of the CX into the LMCA. However, this stent failed to detach from the balloon and was pulled back while still adhered to it, ultimately lodging in the tip of the guiding catheter in a fully expanded, ring-like configuration (Fig. 2D). This was identified as the second major complication.

At this point, due to increasing stress and fatigue of the primary operator, the procedure was handed over to another experienced operator. The guiding catheter was withdrawn and suspended in the ascending aorta. In the same session, a second vascular access was established via the femoral route, and a rapid, coaxial engagement of the left main coronary artery was achieved. The circumflex artery (CX) was rewired using a Sion (Asahi Intecc Co., Japan) guidewire. A 4.0 × 12 mm Xience PRO stent (DES, Abbott Vascular) was successfully implanted in the distal segment of the CX at 18 atm pressure, covering the dissection flap. The proximal part of the previously implanted 3.5 × 48 mm DES in the CX was post-dilated with the balloon of the new stent. Subsequently, a 4.0 × 22 mm Resolute Integrity (Medtronic Inc., Minneapolis, U.S.A.) DES was deployed from the CX into the LMCA at 18 atm pressure. Finally, post-dilation was performed from the LMCA ostium to the distal segment using a 5.0 × 10 mm non-compliant balloon at 24 atm pressure (Fig. 2E). Following these interventions, the patient’s chest pain completely resolved, and successful blood flow was restored in both the LAD and CX (Fig. 2F, Video 1).

**Fig. 3:**
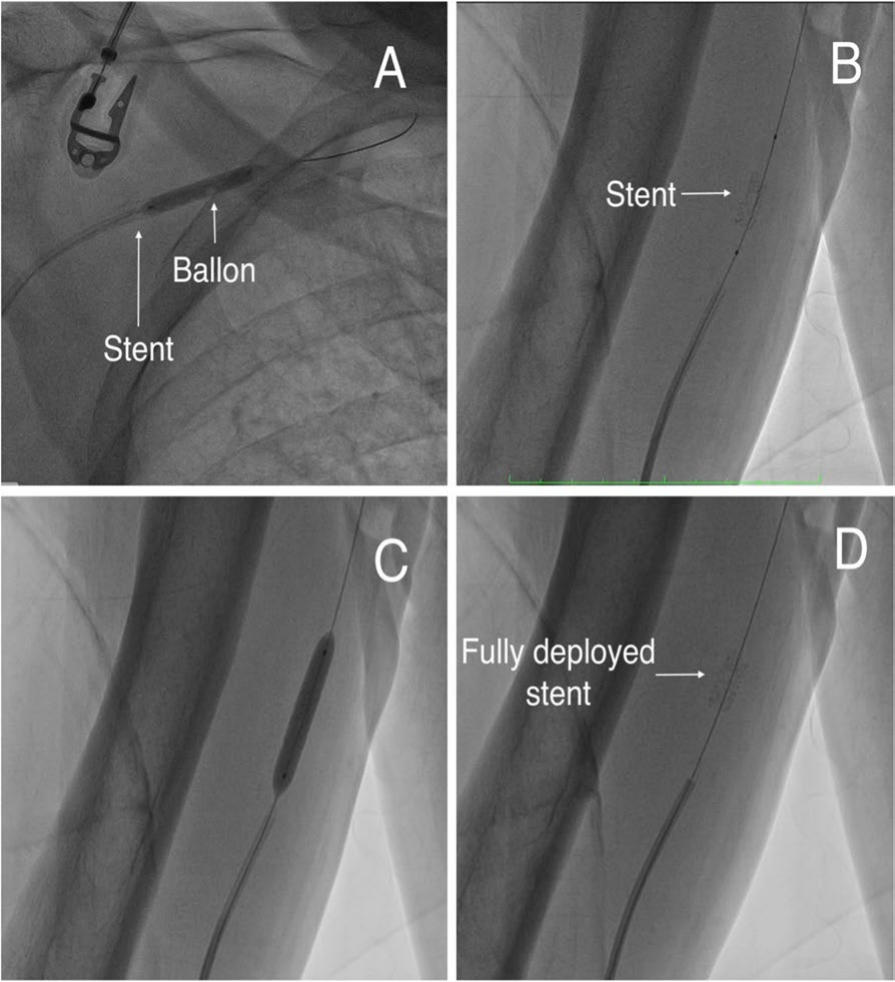
Management of the Dislodged Stent in the Ascending Aorta. **A** Controlled retrieval of the fully expanded stent by gently pulling it back using an inflated balloon positioned within the guiding catheter. **B** Precise alignment of the stent at the level of the right brachial artery using a 4.0 × 22 mm balloon. **C** Deployment of the stent by inflating the balloon at 18 atm to ensure full expansion and secure implantation. **D** Final angiographic view showing the implanted stent in the brachial artery without evidence of embolization or flow limitation

To manage the second complication, attention was directed to the fully expanded stent lodged at the tip of the guiding catheter, which had been suspended in the ascending aorta. A 4.0 × 22 mm balloon was carefully advanced from the radial access through the lumen of the open stent in a deflated state. The balloon was then gently inflated, and the stent-balloon unit was slowly withdrawn under fluoroscopic guidance. To prevent uncontrolled embolization, the open stent was deliberately implanted into the right brachial artery (Fig. 3).

After the intervention, coronary blood flow was preserved in all vessels, and no additional ischemic complications occurred. The patient remained hemodynamically stable and was discharged uneventfully the next day on dual antiplatelet therapy.

**Fig. 4:**
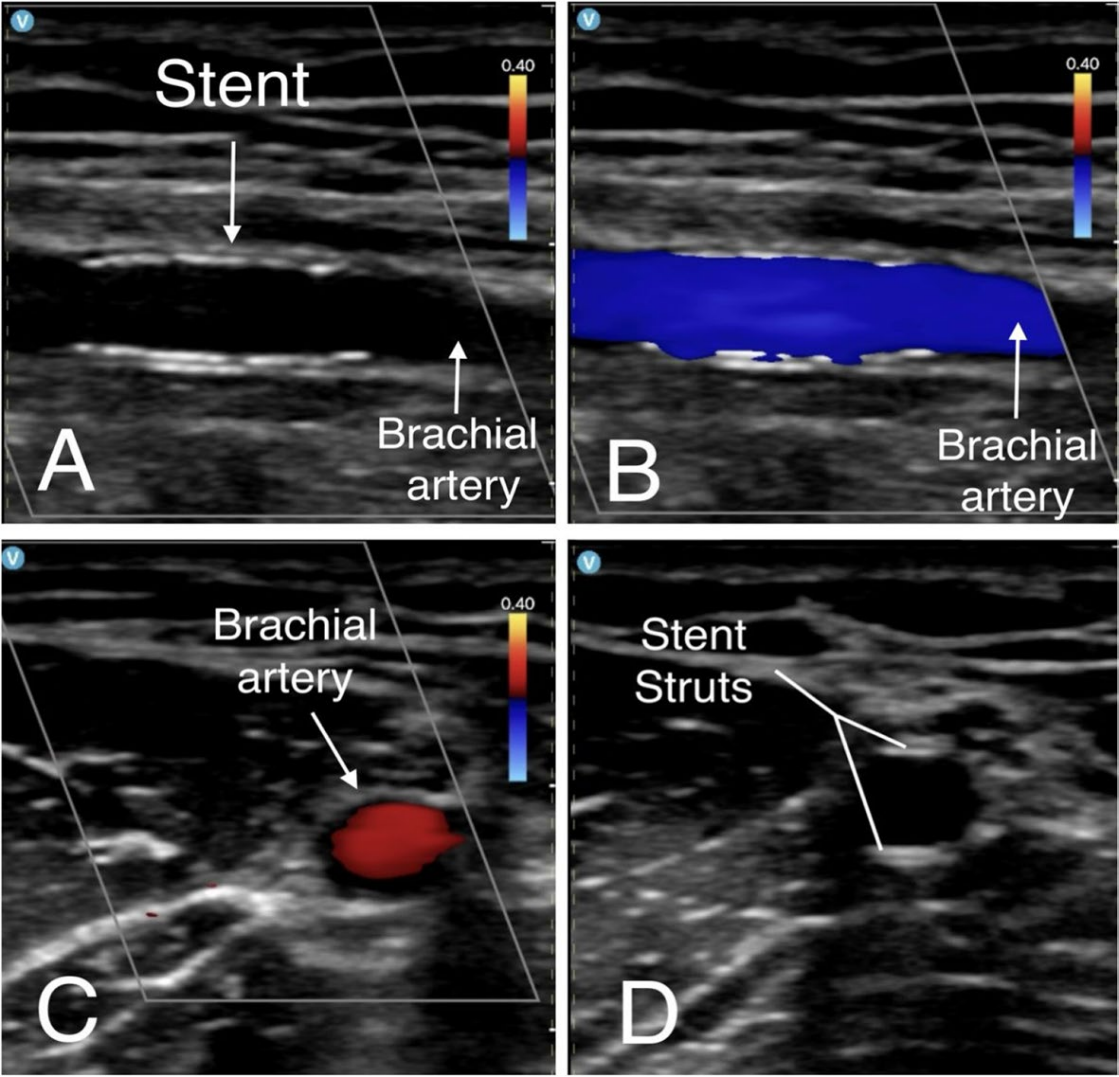
Doppler and ultrasonographic evaluation of the stent implanted in the right brachial artery. **A** Doppler ultrasonography image of the brachial artery (BA) demonstrating optimal apposition of the stent without evidence of in-stent restenosis at the 8-month follow-up. **B** Color Doppler ultrasound confirming preserved luminal flow through the stent, with no signs of turbulence or hemodynamic obstruction. **C** Doppler ultrasound imaging confirms that the vessel in which the stent was deployed is the BA, shown in axial view. **D** High-resolution imaging showing optimal stent strut apposition to the vessel wall, with no malapposition or neointimal hyperplasia. (BA = brachial artery; DES = drug eluting stent)

At the 8-month follow-up, the patient continued to be asymptomatic with sufficient exercise capacity. Doppler and ultrasonographic evaluation of the stent implanted in the right brachial artery revealed no evidence of in-stent restenosis (Fig. 4).

## Dıscussıon

Iatrogenic left main coronary artery (LMCA) dissection is an uncommon but life-threatening complication encountered in the catheterization laboratory, with a reported incidence of 0.07% [4, 6–9]. Several anatomical and procedural factors have been associated with an increased risk of iatrogenic LMCA dissection, including atypical LMCA anatomy or origin, underlying LMCA atherosclerosis, limited operator experience, forceful manual contrast injection, non-coaxial catheter engagement, deep catheter intubation, and subintimal guidewire advancement—particularly when stiffer guidewires are used. In addition, inappropriate guide catheter selection, including the use of extra back-up and Judkins Left catheters, has been reported as a contributing factor [1, 10].

Published case reports and small series indicate that both the clinical presentation and management of iatrogenic LMCA dissection are highly variable. Although conservative treatment has been reported [2, 4] to be successful in hemodynamically stable patients with preserved distal flow, many cases—particularly those associated with hemodynamic instability—require immediate revascularization. Emergent percutaneous coronary intervention with bailout stenting or urgent surgical revascularization have both been described as effective treatment options, even in patients presenting with cardiogenic shock or impending hemodynamic collapse [11, 12].

Although conservative management may be considered in stable patients, the majority require urgent revascularization due to the high risk of hemodynamic collapse [1, 3]. Management decisions are guided mainly by clinical stability, ECG changes, and distal flow [13], and wire position must be secured promptly to prevent procedural failure [9].

In our case, LMCA dissection was likely precipitated by non-coaxial catheter engagement and forceful contrast injection. Rapid recognition and successful wiring allowed immediate stent deployment; however, a second, exceptionally rare complication occurred—migration of a fully deployed stent. While stent loss during PCI has an incidence of 0.3–8.4% [14, 15], complete dislodgement of a fully expanded stent is extremely rare [5].

The mechanism of retrograde migration of the fully deployed stent was evaluated based on procedural findings. As demonstrated in the procedural video, the stent was fully expanded and circumferentially engaged the tip of the guiding catheter in a ring-like configuration, suggesting migration occurred after complete deployment. A plausible explanation is premature withdrawal of the delivery system before complete balloon deflation. In addition, the use of a highly concentrated contrast–saline mixture within the balloon, suboptimal coaxial alignment, and relative undersizing of the stent compared with the angiographically estimated vessel diameter may have reduced frictional resistance and facilitated stent migration.

Percutaneous retrieval is the preferred strategy, most commonly performed using the small-balloon technique with reported success rates up to 70%. Alternative options include loop snares, basket catheters, or crushing the stent against the vessel wall when retrieval fails [15]. However, retrieval strategies for fully expanded stents are inherently limited, with only a few cases described in the literature. Successful retrieval using the twisted wire technique has been reported in isolated cases [16]; however, this approach has failed in other cases [5], requiring alternative management, such as crushing the fully deployed stent against the vessel wall with a second stent within the coronary circulation. In contrast, in our case, the stent migrated outside the coronary system into the ascending aorta, creating a substantial risk of cerebral or systemic embolization [17, 18].

Although the transradial approach offers advantages in safety and patient comfort, it limits the ability to perform retrieval procedures due to smaller catheter diameters [18]. Given the fully expanded 4.0-mm stent diameter and the technical constraints of the transradial approach, intentional implantation into a peripheral artery was considered the safest option to prevent uncontrolled systemic embolization, in line with previously reported cases of deliberate stent implantation in peripheral vessels, such as the iliac artery [19]. In this case, the brachial artery was deliberately selected for its adequate vessel caliber, relatively straight course, and location remote from areas of repetitive flexion or external compression. This strategy minimized the risks of stent deformation, fracture, or flow limitation and eliminated the need for surgical intervention, allowing for easier clinical and ultrasonographic follow-up. The patient was already receiving standard dual antiplatelet therapy due to the coronary drug-eluting stents implanted during the index procedure, which also provided adequate protection against thrombotic complications of the brachial artery stent.

Intravascular imaging modalities such as IVUS or optical coherence tomography (OCT) could have provided a more precise assessment of coronary vessel size and stent apposition, and may have allowed further optimization of coronary stent sizing and deployment strategy [20, 21]; however, the lack of intravascular imaging in our laboratory was a procedural limitation.

Another important aspect of this case was the elevation of troponin levels despite angiographically normal coronary arteries, raising the suspicion of Type 2 myocardial infarction. The combination of doxazosin-induced hypotension and borderline polycythemia (hemoglobin 18.6 g/dL) may have synergistically impaired myocardial oxygen delivery through reduced coronary perfusion and increased blood viscosity [22, 23]. These mechanisms likely explain the biochemical evidence of myocardial injury.

## Conclusion

This case demonstrates that two rare and serious complications can be managed percutaneously. The technique we used, inspired by the small-balloon method, proved to be an effective and feasible solution. When retrieval is unsuccessful, intentional stent implantation into a peripheral artery may be a safe alternative. In similar scenarios, operator experience and optimal use of available resources can lead to successful outcomes.

## Supplementary Information

Supplementary Material 1.

## Data Availability

No datasets were generated or analysed during the current study.

## Abbreviations

CABG: Coronary artery bypass graft
CAG: Coronary angiography
CX: Circumflex artery
DES: Drug-Eluting Stent
eGFR: Estimated glomerular filtration rate
EF: Ejection fraction
LAD: Left anterior descending
NSTEMI: Non-ST elevation myocardial infarction
PCI: Percutaneous coronary intervention

## References

[1] Celik M, Yuksel UC, Yalcinkaya E, Gokoglan Y, Iyisoy A. Conservative treatment of iatrogenic left main coronary artery dissection: report of two cases. Cardiovasc Diagn Ther. 2013;3(4):244–6. 10.3978/j.issn.2223-3652.2013.10.04. 24400208 10.3978/j.issn.2223-3652.2013.10.04 PMC3878113

[2] Awadalla H, Sabet S, El Sebaie A, Rosales O, Smalling R. Catheter-induced left main dissection incidence, predisposition and therapeutic strategies experience from two sides of the hemisphere. J Invasive Cardiol. 2005;17(4):233–6. 15831980

[3] Onsea K, Kayaert P, Desmet W, Dubois CL. Iatrogenic left main coronary artery dissection. Neth Heart J. 2011;19(4):192–5. 10.1007/s12471-011-0089-1. 22020998 10.1007/s12471-011-0089-1 PMC3077877

[4] Eshtehardi P, Adorjan P, Togni M, et al. Iatrogenic left main coronary artery dissection: incidence, classification, management, and long-term follow-up. Am Heart J. 2010;159(6):1147–53. 10.1016/j.ahj.2010.03.012. 20569732 10.1016/j.ahj.2010.03.012

[5] Çetin Şanlıalp S, Tekin I, Şanlıalp M. Dislodgement of the fully expanded stent and the management of this complication by using crushing technique. J Updates Cardiovasc Med. 2020;8(3):157–62. 10.32596/ejcm.galenos.2020.04.019.

[6] Tabet R, Nalluri N, Daneshvar F, et al. (October 04, 2018) Interventional Approach to Left Main Coronary Artery Dissection. Cureus. 10(10):e3410. 10.7759/cureus.3410 ) 10.7759/cureus.3410 PMC6281447 30538898

[7] Boyle AJ, Chan M, Dib J, Resar J. Catheter-induced coronary artery dissection: risk factors, prevention and management. J Invasive Cardiol. 2006;18(10):500–3. 17015916

[8] Amano H, Kubo S, Osakada K, et al. Clinical outcomes and angiographic results of bailout stenting for guide catheter-induced iatrogenic coronary artery dissection - impact of stent type. Circ J. 2020;84(3):1746–53. 10.1253/circj.CJ-20-0123. 32893259 10.1253/circj.CJ-20-0123

[9] Page E, Kostantinis S, Karacsonyi J, et al. Incidence, treatment and outcomes of coronary artery dissection during percutaneous coronary intervention. J Invasive Cardiol. 2023;35(7):E341–54. 10.25270/jic/23.00007. 37769612 10.25270/jic/23.00007

[10] Hentati M, vd. Management and prognosis of catheter induced aortocoronary dissection: A multicentric observational study. Arch Cardiovasc Dis Suppl., c. 14, sy 1, ss. 9–10, Oca. 2022, 10.1016/j.acvdsp.2021.09.014.

[11] Ozdol C, Oral D, Tutar E. Catheter-induced left main coronary artery dissection resulting in abrupt closure and cardiac arrest: successful stenting during resuscitation. J Invasive Cardiol. 2007;19(4):E93–5. 17404412

[12] Connors JP, Thanavaro S, Shaw RC, Sandza JG, Ludbrook PA, Krone RJ. Urgent myocardial revascularization for dissection of the left main coronary artery: a complication of coronary angiography. J Thorac Cardiovasc Surg. 1982;84(3):349–52. 6981034

[13] Jeyakumaran B, Raj A, Pandit BN, Kumar T, Deora S. Iatrogenic left main coronary artery dissection due to pin-hole balloon rupture: not to be panicked…. Acute Card Care. 2015;17(4):80–2. 10.3109/17482941.2016.1174271. 27283143 10.3109/17482941.2016.1174271

[14] Eggebrecht H, Haude M, von Birgelen C, et al. Nonsurgical retrieval of embolized coronary stents. Catheter Cardiovasc Interv. 2000;51(4):432–40. 10.1002/1522-726x(200012)51:4%3c432::aid-ccd12%3e3.0.co;2-1 . 10.1002/1522-726x(200012)51:4<432::aid-ccd12>3.0.co;2-1 11108675

[15] Brilakis ES, Best PJ, Elesber AA, et al. Incidence, retrieval methods, and outcomes of stent loss during percutaneous coronary intervention: a large single-center experience. Catheter Cardiovasc Interv. 2005;66(3):333–40. 10.1002/ccd.20449. 16142808 10.1002/ccd.20449

[16] Hsu PC, Lin TH, Lee WH, Sheu SH. Inadvertent extraction of a deployed stent after using twisted wire technique. Kaohsiung J Med Sci. 2014;30(1):55–6. 10.1016/j.kjms.2013.09.007. 24388061 10.1016/j.kjms.2013.09.007 PMC11916555

[17] Alomar ME, Michael TT, Patel VG, et al. Stent loss and retrieval during percutaneous coronary interventions: a systematic review and meta-analysis. J Invasive Cardiol. 2013;25(12):637–41. 24296383

[18] Kwan TW, Chaudhry M, Huang Y, et al. Approaches for dislodged stent retrieval during transradial percutaneous coronary interventions. Catheter Cardiovasc Interv. 2013;81(6):E245–9. 10.1002/ccd.24483. 22581524 10.1002/ccd.24483

[19] Meisel SR, DiLeo J, Rajakaruna M, Pace B, Frankel R, Shani J. A technique to retrieve stents dislodged in the coronary artery followed by fixation in the iliac artery by means of balloon angioplasty and peripheral stent deployment. Catheter Cardiovasc Interv. 2000;49(1):77–81. 10.1002/(sici)1522-726x(200001)49:1%3C;77::aid-ccd17%3E;3.0.co;2-y. 10.1002/(sici)1522-726x(200001)49:1<77::aid-ccd17>3.0.co;2-y 10627373

[20] Zhang J, Gao X, Kan J, et al. Intravascular ultrasound versus angiography-guided drug-eluting stent implantation: the ULTIMATE trial. J Am Coll Cardiol. 2018;72(24):3126–37. 10.1016/j.jacc.2018.09.013. 30261237 10.1016/j.jacc.2018.09.013

[21] Ali ZA, Maehara A, Généreux P, et al. Optical coherence tomography compared with intravascular ultrasound and with angiography to guide coronary stent implantation (ILUMIEN III: OPTIMIZE PCI): a randomised controlled trial. Lancet. 2016;388(10060):2618–28. 10.1016/S0140-6736(16)31922-5. 27806900 10.1016/S0140-6736(16)31922-5

[22] Chapman AR, Taggart C, Boeddinghaus J, Mills NL, Fox KAA. Type 2 myocardial infarction: challenges in diagnosis and treatment. Eur Heart J. 2025;46(6):504–17. 10.1093/eurheartj/ehae803. 39658094 10.1093/eurheartj/ehae803 PMC11804249

[23] Tahiraj X, Bakalli A, Krasniqi X, Çitaku H, Krasniqi F, Koçinaj D. Acute myocardial infarction and polycythemia rubra vera: the double effect of treatment with hydroxyurea. Radiol Case Rep. 2024;19(8):3386–9. 10.1016/j.radcr.2024.05.014. 10.1016/j.radcr.2024.05.014 PMC11140144 38827039

